# Predator Diet and Trophic Position Modified with Altered Habitat Morphology

**DOI:** 10.1371/journal.pone.0147759

**Published:** 2016-01-29

**Authors:** Alexander Tewfik, Susan S. Bell, Kevin S. McCann, Kristina Morrow

**Affiliations:** 1 Department of Integrative Biology, University of South Florida, Tampa, Florida 33620-5200, United States of America; 2 Department of Integrative Biology, University of Guelph, Guelph, Ontario, Canada; Texas A&M University at Galveston, UNITED STATES

## Abstract

Empirical patterns that emerge from an examination of food webs over gradients of environmental variation can help to predict the implications of anthropogenic disturbance on ecosystems. This “dynamic food web approach” is rarely applied at the coastal margin where aquatic and terrestrial systems are coupled and human development activities are often concentrated. We propose a simple model of ghost crab (*Ocypode quadrata*) feeding that predicts changing dominant prey (*Emerita talpoida*, *Talorchestia* sp., *Donax variablis*) along a gradient of beach morphology and test this model using a suite of 16 beaches along the Florida, USA coast. Assessment of beaches included quantification of morphological features (width, sediments, slope), macrophyte wrack, macro-invertebrate prey and active ghost crab burrows. Stable isotope analysis of carbon (^13^C/^12^C) and nitrogen (^15^N/^14^N) and the SIAR mixing model were used to determine dietary composition of ghost crabs at each beach. The variation in habitat conditions displayed with increasing beach width was accompanied by quantifiable shifts in ghost crab diet and trophic position. Patterns of ghost crab diet were consistent with differences recorded across the beach width gradient with respect to the availability of preferred micro-habitats of principal macro-invertebrate prey. Values obtained for trophic position also suggests that the generalist ghost crab assembles and augments its diet in fundamentally different ways as habitat morphology varies across a highly dynamic ecosystem. Our results offer support for a functional response in the trophic architecture of a common food web compartment (ghost crabs, macro-invertebrate prey) across well-known beach morphologies. More importantly, our “dynamic food web approach” serves as a basis for evaluating how globally wide-spread sandy beach ecosystems should respond to a variety of anthropogenic impacts including beach grooming, beach re-nourishment, introduction of non-native or feral predators and human traffic on beaches.

## Introduction

Ecologists have long searched for general topological patterns in food webs across disparate ecological systems with the goal of identifying structure that mediates the stability of nature’s complex networks [1–4]. This approach looks for constant patterns in common food web metrics (e.g., number of trophic links) using a “static food web approach”. While such efforts have produced a powerful and informative literature, an emerging body of research, instead, considers how a specific food web (e.g., lake, rocky intertidal, sandy beach, mudflat) changes, or adapts, across environmental/physical gradients [5–8]. This latter “dynamic food web approach” offers the potential to identify how ecosystems generally respond to environmental variation and disturbance not only in space but also in time.

Researchers utilizing the dynamic food web approach have employed stable isotopes to show that changes in food web structure appear to be most dramatically driven by mobile consumers of a high trophic level, which are capable of behaviorally responding to spatial variation in prey (i.e., resource availability) and environmental conditions (i.e., resource accessibility). For example, the degree to which cold water-adapted Lake trout consume prey from warmer nearshore habitats (versus the cold pelagic habitat they prefer) has been hypothesized to decrease with increased near shore temperatures [9, 10]. This mechanism suggests that cold water adaptation of trout makes the near shore zone, more or less accessible, depending on temperature. Clearly though, increased prey densities (i.e., resource availability) in different habitats can also influence the foraging decisions of key mobile and upper trophic level consumers [11, 12]. Importantly, when such documented changes in food web structure are mechanistically understood, they can also act as powerful tools to predict the implications of altered resource accessibility or availability which is often the consequence of human impact on ecosystems.

### A Model System: Sandy Beaches at the Terrestrial-Marine Interface

While researchers have begun to find consistent patterns in ecosystem responses to change in habitat, disturbance regimes and resources [7, 12], the breadth of ecosystems studied remains limited. Most dynamic food web studies have focused upon either aquatic or terrestrial ecosystems, but few have considered organisms that “couple” across these two major ecosystem types. Sandy beaches, the most common habitat along continental margins and bordering 70% of all marine coasts [13–15], provide an interesting model system to examine general food web dynamics of coupled aquatic-terrestrial systems [5, 15, 16]. They often support a key mobile predator of high trophic status (e.g., Ghost crab, *Ocypode* sp.) and several dominant primary consumers (mole crabs, coquina clams, amphipods).

**Fig 1:**
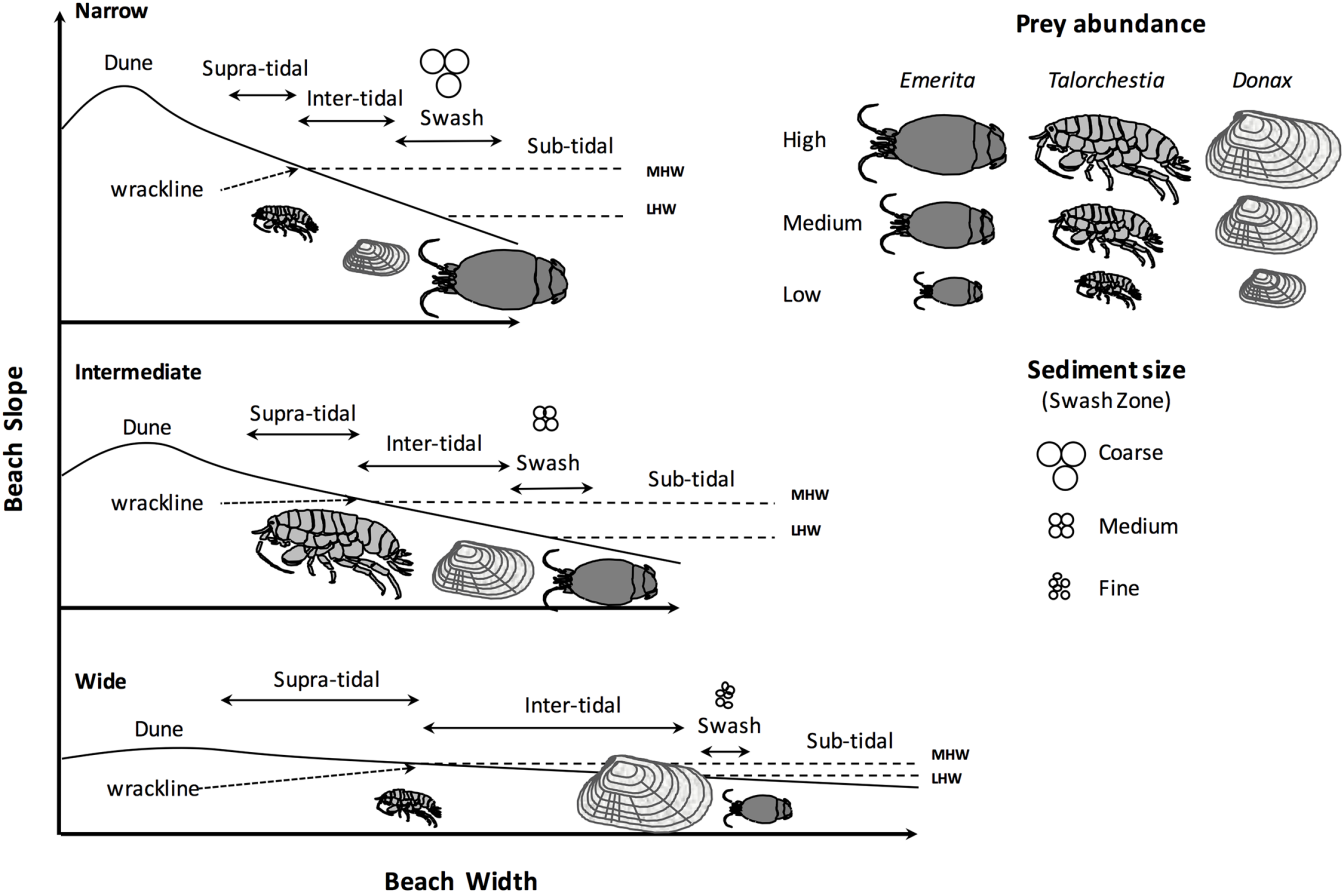
Schematic of relative changes in beach morphology with increasing width (x-axis, one order of magnitude) and decreasing slope (y-axis, one order of magnitude) including the distribution of micro-habitat compartments (sub-tidal, swash, inter-tidal, supra-tidal zones), macrophytes (wrackline), principal macro-invertebrate primary consumers and relative abundance (mole crab—*Emerita talpoida*, amphipod—*Talorchestia* spp., coquina calm—*Donax variablis*) and associated relative swash sediment size (coarse, medium, fine) and water levels (MHW = mean high water, MLW = mean low water). Mean individual prey sizes range from 1.0 (amphipod) to 2.5 (mole crab) cm in length. The mole crab (*E*. *talpoida*) passively filters particulate organics from receding waves in the swash zone and maximizes feeding efficiency at exposed beaches characterized by large waves, steep slopes and strong wave surge [57, 79, 80]. The coquina clam (*D*. *variabilis*) is also found in the swash zone but appears to prefer lower wave energy sites while actively filtering organic particulates [57, 79, 80]. Semi-terrestrial amphipods (*Talorchestia* spp.) occupy the low supra-tidal areas of sandy beaches in strong association with the spray zone and damp stranded macrophyte materials where they can feed and shelter [57, 84].

Sandy beach communities are influenced by several environmental parameters including, wave climate, tidal regime and sediment source which interact to create a gradient of beach morphology [5, 17–21]. Ecosystem size has served as a major axis along which dynamic food web structure has been examined [2, 22, 23]. Correspondingly, beach width, as an proxy for ecosystem size, has been proposed to correlate with a number of important physical attributes that likely impact resource accessibility and availability in sandy beach systems [5, 24–26]. Here we refer to beach morphology more broadly as the set of habitat features that includes ecosystem size (i.e., beach width) as well as other physical qualities (e.g., substrate type, slope) that may influence broader community structure [9, 27, 28]. In the most basic sense, sandy beach morphologies typically range from steep and narrow with coarse grain sediments (i.e. reflective) to flat and wide with an increasing proportion of fine grain sediments (i.e. dissipative) [19–21]. This generates a suite of intertidal to supra-tidal conditions which explains the distribution of organisms within and between beach types and thus influence associated resource accessibility and availability to higher order consumers [5, 13, 24] (Fig 1). Additionally, food subsidies provided by accumulated macrophyte wrack that originates offshore and is then transported to the coast [29–31], may be strongly influenced by beach morphology that directly determines the depositional position and quality of wrack resources [32]. As a result, contrasting patterns of abundance and spatial distribution of wrack across the beach may further contribute to variability of resource accessibility and availability (Fig 1) [33–35].

### A principal food web compartment: Ghost crabs and macro-invertebrate prey

Evaluation of variation of food webs over environmental gradients typically requires examination over a large geographic area encompassing the principal trophic interactions. Variation in beach morphology is well documented along the 2170 km coast of Florida, USA and information on beach food webs suggest the common occurrence of a semi-terrestrial ocypodid crab as an important secondary consumer inhabiting these beaches and more generally, worldwide [36–41]. The vast majority of the Atlantic ghost crab’s (*Ocypode quadrata*) life history and foraging activities straddle two distinct micro-habitats, the aquatic shallow intertidal zone (i.e., swash zone) and terrestrial supra-tidal beach [38], which vary with sandy beach morphology. Within the latter suitable (damp) macrophyte material may be stranded thereby forming a wrackline which provides food and reduces desiccation stress for selected macro-invertebrate prey and allows ghost crabs to access these edible resources [37, 39, 41–43] (Fig 1).

The Atlantic ghost crab has been described as both a predator and scavenger with its diet thought to consist primarily of swash-zone inhabiting mole crabs (*Emerita talpoida*) and coquina clams (*Donax variabilis*), as well as semi-terrestrial amphipods (*Talorchestia* spp.) which utilize damp wrack in the supratidal [36, 37, 44, 45] (Fig 1). All three principal prey taxa provide critical trophic links in sandy beach ecosystems globally but have distinct feeding modes and preferred micro-habitats [33, 46, 47]. Ghost crab diets may be opportunistically supplemented by marine carrion, and/or microphagous depositing feeding- either of which may modify ghost crab trophic position, *sensu* [38, 48], albeit such resources most likely contribute only a small percentage to their diet [37, 49, 50].

Although ghost crab diets have been described from several locations, little information exists on the variation in their diets that may be expected across the broad spectrum of beach morphology despite the ghost crab’s wide distribution and importance to the function of sandy beach ecosystems [37, 42, 44, 51, 52]. In lakes, modification of prey populations, predator diets, trophic position and degree of omnivory has been observed along gradients of lake morphology reflecting differences in predator access to particular food web compartments supporting important prey [9, 27]. Similarly, in sandy beaches, based upon known distribution and preferred habitats of the main prey of ghost crabs (see Fig 1) (mole crabs, coquina clams, and semi-terrestrial amphipods) [5] and invertebrate community composition [17, 40, 53], the relative availability of the most commonly accessed prey change in response to altered beach width, substrate type and slope. Thus the gradient of sandy beach morphologies may be viewed as a series of micro-habitat compartments (swash zone, wrackline) varying in prey accessibility and availability which should have implications for links to higher trophic level consumers (i.e. ghost crab) (Fig 1).

In what follows, we broaden an earlier examination of the diets of the widely distributed Atlantic ghost crab, *Ocypode quadrata*, [43] to encompass 16 barrier island beaches along the sub-tropical Gulf of Mexico and Atlantic coasts of the United States and make predictions about the changing structure of food webs that accompany the gradient of physical attributes of the surveyed beaches. Identifying conspicuous changes in the conversion of carbon and nitrogen by a major predator—the ghost crab—across a range of sandy beach morphology is a critical first step in describing food web configurations and their variation over time and space [10, 27, 47, 54].

**Fig 2:**
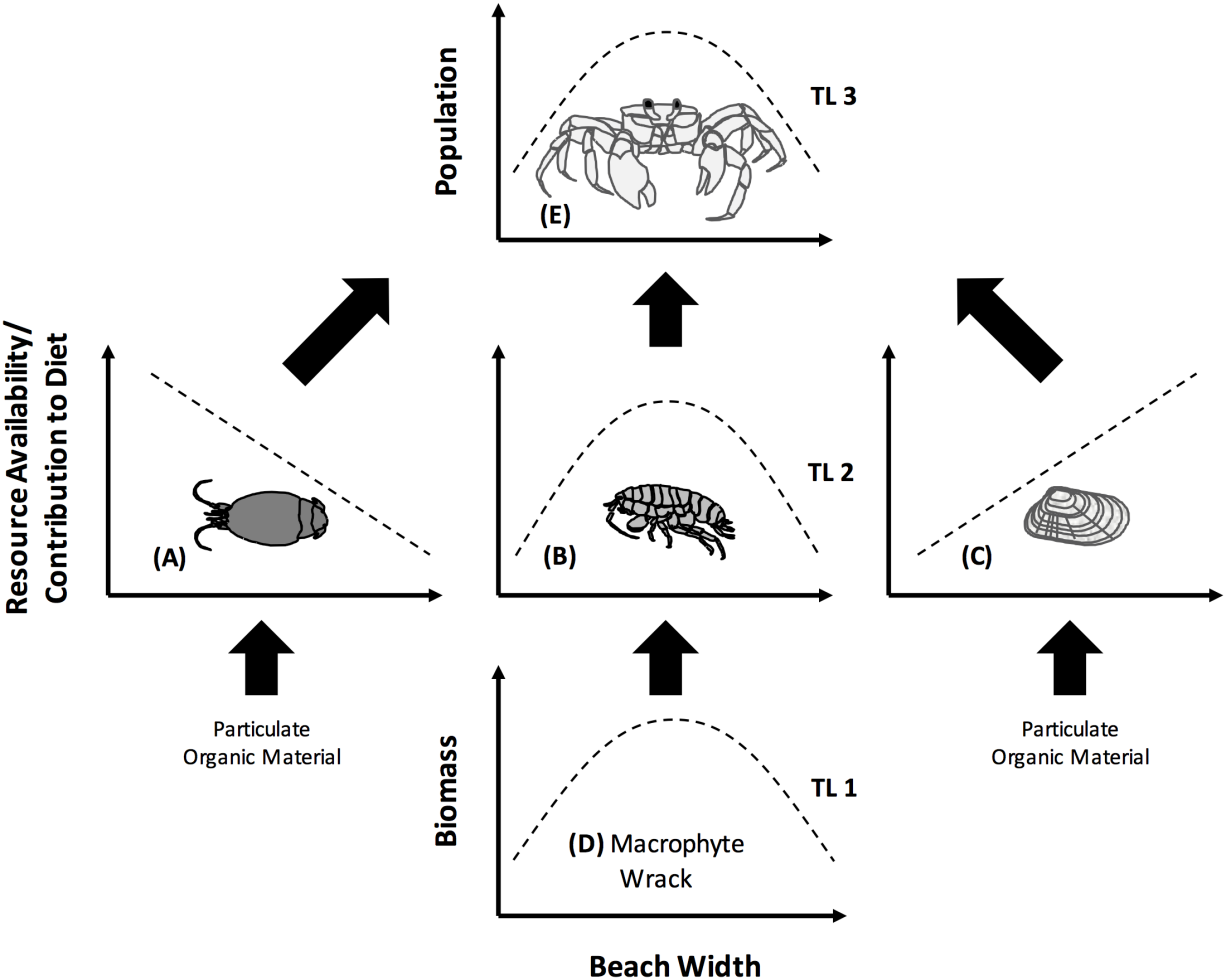
A working model reflecting relative measures of resource availability/contribution to diets (y-axis) of a secondary consumer (ghost crab, *Ocypode quadrata*) with increasing beach width (x-axis). As beach width increases the preferred habitat conditions for the principle macro-infaunal prey (A = mole crab—*Emerita talpoida*, B = amphipod—*Talorchestia* spp., C = coquina calm—*Donax variablis*) shift from high energy, coarse sediment beaches preferred by mole crabs (A) to low energy, finer sediment beaches preferred by coquina clams (C). At intermediate beach width the accumulation of damp macrophyte wrack (D) provides both shelter and food for semi-terrestrial amphipods (B), thereby supporting a food subsidy for ghost crabs. Abundance of *Ocypode quadrata* (E) may be highest at intermediate width beaches if multiple resources are highly accessible for the consumer. Arrows indicate energy flow direction only; relative uses of prey resources (A, B, C) are expected to fluctuate across beach width. Aquatic-derived particulate organic material provides primary resources to mole crab and coquina clams. TL = trophic level.

We propose a simple model of ghost crab feeding along a gradient of beach morphology (Fig 2). We use beach width as our principal metric of habitat morphology as it best integrates a number of physical parameters of the sandy beach habitat where ghost crabs live including beach slope, median sediment grain size, and availability and quality of macrophyte wrack [43, 55, 56]. We predict that diet composition of ghost crabs will vary over beach width mainly reflecting prey availability as preferred micro-habitats and distinct feeding modes of each prey (mole crab, coquina clam, amphipod) vary as described earlier (Figs 1 and 2). Ghost crabs are highly mobile so prey accessibility is not considered to be a major factor in our working model. Assuming constant mortality of ghost crabs over the gradient of resource availability we further anticipate that ghost crab population abundance will be elevated at intermediate beach width sites (Fig 2). Here both principal swash prey (mole crab, coquina clam) and elevated populations of semi-terrestrial amphipods, associated with localized deposition of suitable macrophyte wrack subsidies (Fig 1), are all available for consumption allowing enhanced survival and growth of ghost crabs (Fig 2). Based on our model, the trophic position of ghost crabs is predicted to be similar across the range of beach widths. The main sources of the ghost crab diet are all primary consumers which often use macrophyte materials, particulate in the swash zone or as detritus in the wrackline, as their main source of nitrogen [33, 47]. Thus the trophic position of the predator should remain relatively unchanged regardless of what combination of the three principal prey are consumed. However, some degree of omnivory, scavenging of carrion or microphagous depositing feeding, may lead to altered trophic positions of ghost crabs [11, 38, 49].

## Methods

### Field sampling

**Fig 3:**
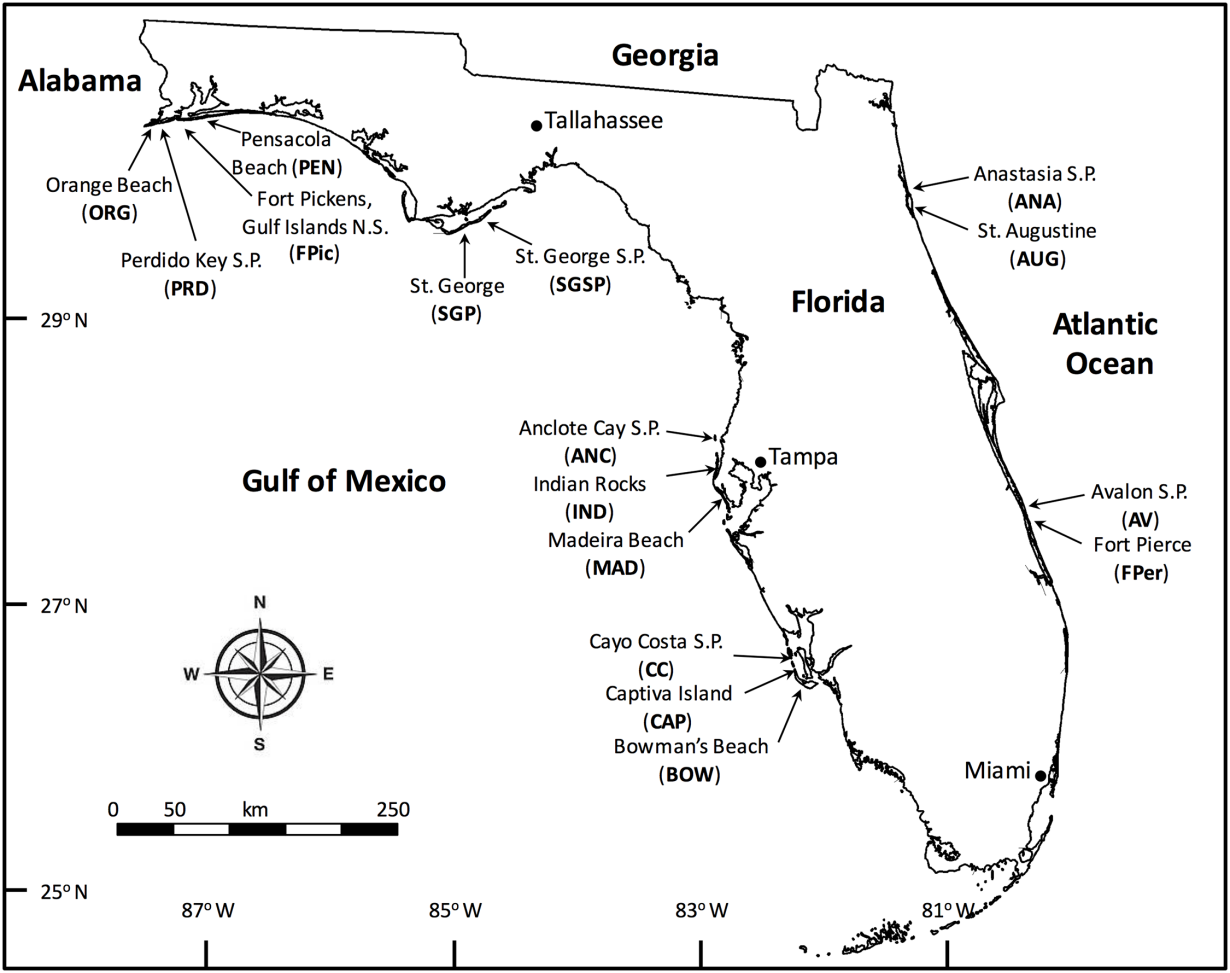
Geographic distribution of barrier island beach sites (N = 16, S1 Table) sampled during this study (2010–2012). S.P. = State Park, N.S. = National Seashore.

Sixteen sub-tropical beaches across a range of beach morphologies were investigated along the Gulf of Mexico and Atlantic Ocean (Fig 3). Work was conducted with a Special Activity License permit (10-1220C-SR), Florida Fish and Wildlife Conservation Commission and a US National Parks Service collection permit (GUIS-2010-SCI-0023) for the Gulf Islands National Seashore. All sites sampled had linear, smooth coastlines characterized by well-developed beaches located on barrier islands with varying levels of human development. However, all beaches were undeveloped from the fore-dune down to the low tide, with all sites capable of supporting swash zone macro-fauna, wrack dwelling amphipods and ghost crabs. Sampling was conducted on six occasions (May/June 2010, July 2010, September 2010, November 2010, April 2011 and July 2012), avoiding the coldest months (December–March) when beach swash macrofauna are least active and have reduced availability as they move offshore, semi-terrestrial amphipods move into sediments and ghost crabs remain in their burrows [57–59]. Mean values were calculated for all parameters across all time series sampled.

The general sampling protocol involved the haphazard placement of three, 4 m wide belt transects, separated by 50 m, across the width of each of the 16 beaches (top of the swash to base of the dune) within 2 h of low tide. For each beach, morphological assessments included measurements of beach width (n = 3), foreshore slope (n = 9, digital clinometer at the mid- swash) and median sediment grain size of swash zone (n = 9, 60 ml core to 10 cm depth) during May/June 2010, September 2010, April 2011 and July 2012. Additionally, macrophytes were assessed from nine 625 cm^2^ quadrats collected along the wrackline during May/June, September 2010 and July 2012. Sediment cores were returned to the laboratory, dried for 24 h at 60°C and analyzed for grain size class distribution using six standard sieves (>2 mm to < 0.125 mm). Wrack samples were returned to the laboratory, examined for the presence of semi-terrestrial amphipods and separated into major components (i.e., macroalgae, seagrass, terrestrial plants, and “other”, including animal and artificial materials) and dried for 24–48 h at 60°C to determine mass (g).

Coquina clams and mole crabs were sampled at each beach within the mid-swash zone using 3 cores (10 cm depth; 10 cm diameter) for each of 3 belt transects (N = 9 cores total) during May/June 2010, September 2010 and July 2012. The contents of each core were sieved (1 mm) in the field and were used to provide an overall assessment of swash dwelling macro-invertebrates. All coquina clams and mole crabs retained on the sieve were placed on ice, brought back to the laboratory and stored at −4°C for subsequent enumeration, tissue extraction and isotopic analysis. The direct enumeration of nocturnally active, semi-terrestrial amphipods in wrack at most sites was difficult [60]. However, given the strong correlation between semi-terrestrial amphipod abundance and wrack, our quantification of wrack biomass is a reasonable proxy for relative amphipod abundance [31, 33, 34].

All active (i.e., evidence of recent excavation) ghost crab burrows within transects were enumerated during all six sampling periods at all sites, with the exception of SGSP and SGP (April 2011 and July 2012) and ANA, AUG, AV and FPer (July 2012), to estimate ghost crab density (# individuals/100 m^2^). Burrow density is considered a reasonable proxy of relative abundance across sites [37, 56] despite any differences to absolute numbers [61]. Ghost crabs were sampled after dusk using nets, placed on ice and stored at −4°C in the laboratory for isotopic analysis.

### Stable Isotope & Dietary Analyses

Stable isotope analysis (SIA) of carbon (δ^13^C) and nitrogen (δ^15^N) was determined for ghost crabs, their dominant macrofaunal prey (mole crabs, amphipods, coquina clams) and basal resources within macrophyte wrack [43, 47, 62–64] from materials collected during May/June 2010, September 2010 and April 2011. Replication of isotopic measurements ranged between 2 and 9 samples with some individual samples incorporating up to 30 individual animals in order to yield sufficient material. Sites with mixed wrack had several dominant materials analyzed for stable isotope signatures and mixed proportionally to provide the overall basal resource signatures. Muscle tissue was extracted from ghost crab (35–45 mm carapace width, adults) walking legs, dried at 60°C for 24 h and ground into a powder for subsequent analyses. All mole crabs and wrack-associated amphipods were washed with 10% HCl solution for 1 min to remove any CaCO_3_ sand particles. The muscle tissue of coquinas was removed from the shell and rinsed with distilled water. Mole crab, amphipod and coquina clam materials were then ground into a powder. Wrack materials were rinsed in distilled water and ground to a powder using a Wiley mill.

Stable isotope measurements were made at the University of South Florida Stable Isotope Laboratory, using a Costech ECS Elemental Analyzer with a ‘zero-blank’ autosampler connected to a Thermo Fisher Scientific (Finnigan) ΔV 3 k eV isotope ratio mass spectrometer. Measured ^13^C/^12^C and ^15^N/^14^N ratios were reported as δ^13^C and δ^15^N values in ‰ relative to the standards, Vienna PeeDee Belemnite carbon and air nitrogen, respectively. The common δ notation is used: δ^13^C or δ^15^N = [(Rsample/Rstandard) − 1] × 1000, where R is, respectively, ^13^C/^12^C or ^15^N/^14^N. Between-sample variation was ±0.1‰ based upon a B-2155 protein certified standard.

The mixing model SIAR, Stable Isotope Analysis in R [65], was used to determine dietary composition of ghost crabs collected from each beach. The SIAR model is a Bayesian mixing model that allows the user to incorporate variability into the sources as well as a trophic enrichment factor (TEF) [65, 66]. We utilized the ‘siarsolomcmcv4’ version of the SIAR model for isotope data with only a single target organism per group [43]. Analyzing the data in this way allowed comparison of individual ghost crabs as well as overall trophic differences among the 16 beach sites. No TEF adjustment was made for carbon as isotopic ratios (δ^13^C) exhibit little (< 1 ‰) to no ^13^C enrichment [63, 67, 68]. The δ^15^N TEF was determined by averaging the differences between our principal consumer (i.e., ghost crabs) and its three principal food sources (mole crab, coquina clam, amphipod) at each site. Site-specific corrections were used because C:N ratio of diets as well as other sources of variation may yield important differences in δ^15^N fractionation [69–71]. Standard deviations for δ^15^N TEF were calculated similarly. The SIAR model was run for 100 000 iterations, dropping the first 10 000 iterations and thinning results by 15, giving a final total of 6000 results. Each of the 6000 results provided a potential model for each individual ghost crab from each site. These 6000 results were then averaged to provide a mean proportion of each food source incorporated into the diet of an individual crab [43].

There were difficulties in obtaining amphipod tissue for stable isotope signatures at sites with low or dry wrack, which is unsuitable habitat for semi-terrestrial amphipods, and at sites not assessed during peak nocturnal activity periods for amphipods. We therefore estimated the amphipod stable isotope values for the mixing model input at these sites using (1) the basal carbon (^13^C) signature of the dominant or proportional mixture of wrack material (S1 Table), considering no trophic fractionation and (2) the resulting nitrogen (^15^N) value based on a linear regression model (y = 1.433x + 2.242, R^2^ = 0.831, P < 0.001) between the dominant or proportional mixture of wrack material (n = 9) and amphipod (n = 9) signatures at sites where both materials were available (ANC, CC, SGSP) similar to previous approaches [68].

An estimate of trophic position (TP) [6] was calculated for each ghost crab population (i.e., site) using the following equation: TP_GC_ = [(δ^15^N_GC_− δ ^15^N_RES_)/2.3] + 2, where δ^15^N_GC_ represents the mean signature (N = 3–9) of ghost crabs; δ^15^N_RES_ represents the weighted mean signature of the three principal resources; and 2.3 is one trophic level change in aquatic environments [6, 72]. Resource δ^15^N value is a sum of the products of dietary component proportion from stable isotope analysis in R (SIAR, see below) and site specific δ^15^N for each principal prey, e.g. [(δ^15^N mole crab = (5.3 * 0.325) + δ^15^N amphipod = (3.8 * 0.325) + δ^15^N coquina = (5.9 * 0.350) = δ^15^N_RES_ = 5.04]. This approach standardizes for the baseline δ^15^N, controls for different mean dietary mixtures and unpredictable differences in assimilation of 1) primary prey resources and/or 2) small proportions of ghost crab resources (carrion, benthic diatoms) not measured at each beach.

### Statistical Analyses

The relationships between the mean proportion of each of the three diet components and the physical parameters (beach width, foreshore slope, median sediment grain size and wrack biomass) across all sites were examined using least squares forward stepwise regression analysis within the statistics module of Sigmaplot version 13.0. Stepwise regression selects independent variables for a multiple linear regression equation from a list of candidate variables to avoid using extraneous variables in the model. The diet component proportions were first transformed (√ArcSine) to address the potential problems of constraints in the covariance, correlation structure and subsequent interpretation linked to the use of compositional data [73]. For those relationships where stepwise linear regression did not yield significant results and exhibited non-linear tendencies to the structure of the data, appropriate non-linear models were applied using individual physical parameters. Stepwise regression analysis was also used to investigate the relationship between ghost crab density and beach physical parameters (mean beach width, foreshore slope, median sediment grain size and wrack biomass). Mean burrow density, as the proxy for ghost crab abundance, was transformed (Log_10_) to meet assumptions of normality [74]. Stepwise regression analysis was also used to examine the relationships between ghost crab trophic position and beach physical parameters. Finally, linear and non-linear models were applied to relationships between proportions of the diet (mole crab, amphipod, coquina clam) and ghost crab trophic position.

## Results

**Fig 4:**
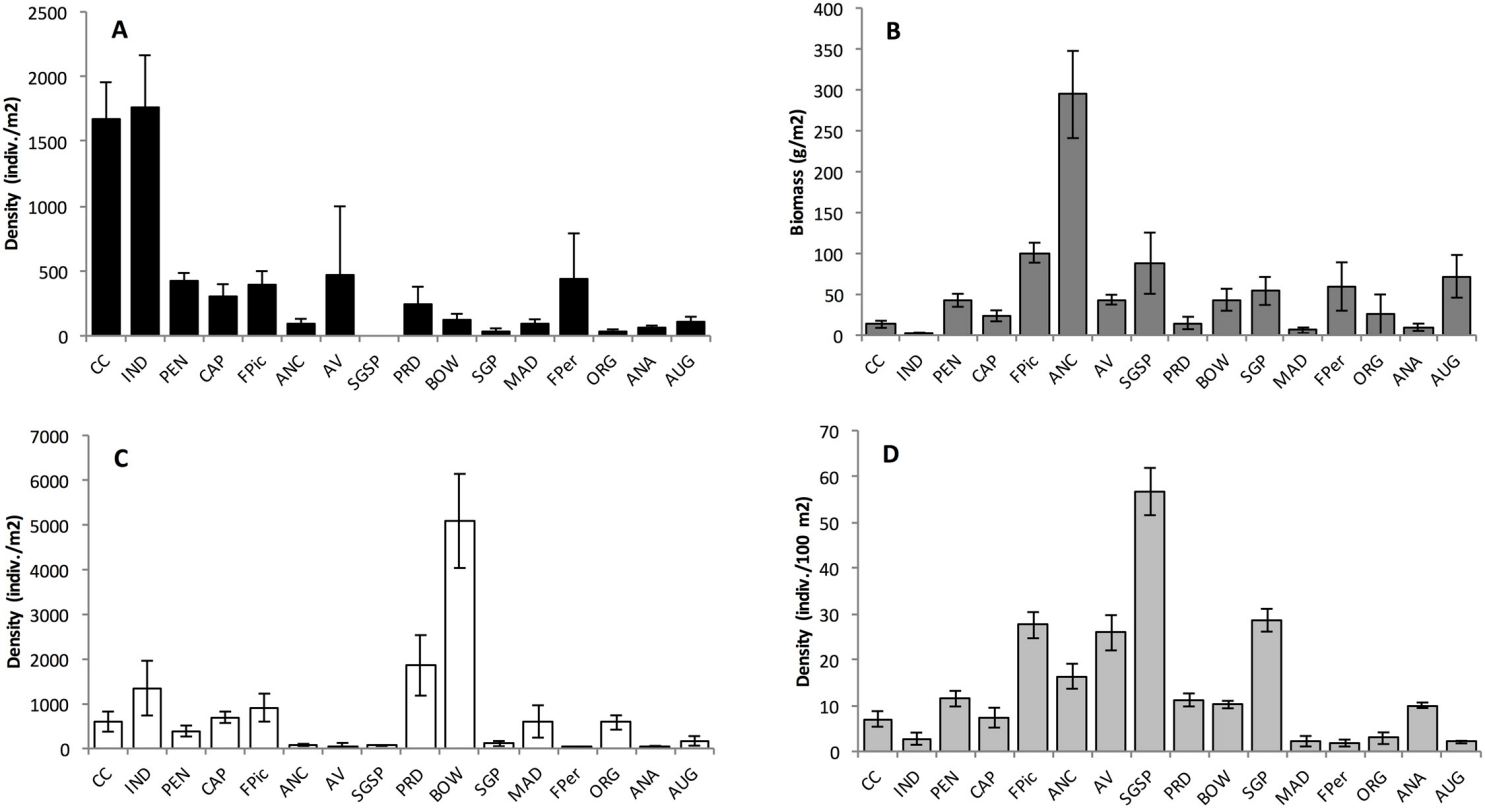
Mean density (+/- se) for A) Mole crab (*Emerita talpoida)*; B) macrophyte wrack; C) Coquina clam (*Donax variablis)*, and D) Ghost crab (*Ocypode quadrata*) in relation to beach width (Fig 2 and S1 Table).

Assessments of beach morphology revealed extensive variation across sites. Mean beach width ranged from 15.7 to 106.6 m, median sediment grain size ranged from 0.1875 mm (fine sand, 0.125–0.5 mm) to 1.5 mm (very coarse sand, 1.0–2.0 mm), and foreshore slope ranged between 2.4 and 17.2 degrees across sites (S1 Table). Abundance of mole crabs, macrophyte wrack, coquina clams and ghost crabs also varied considerably across sites (Fig 4), with mole crabs and coquina clams the only macro-invertebrate prey observed in swash cores. Highest abundances of each of the three prey resources, using wrack as a proxy for semi-terrestrial amphipods, occurred at beaches of different width (Fig 4). Mole crab density was highest at the two narrowest sites (CC, IND), macrophyte wrack biomass was highest at two intermediate width sites (ANC, SGSP) and coquina clam density was highest at two of the wider intermediate sites (PRD, BOW) (Fig 4). Highest ghost crab densities were recorded from beaches of intermediate width (29 to 49 m, FPic to SGP) but were comparatively lower at both narrow (< 29 m) and wide (> 49 m) beaches (Fig 4d).

**Fig 5:**
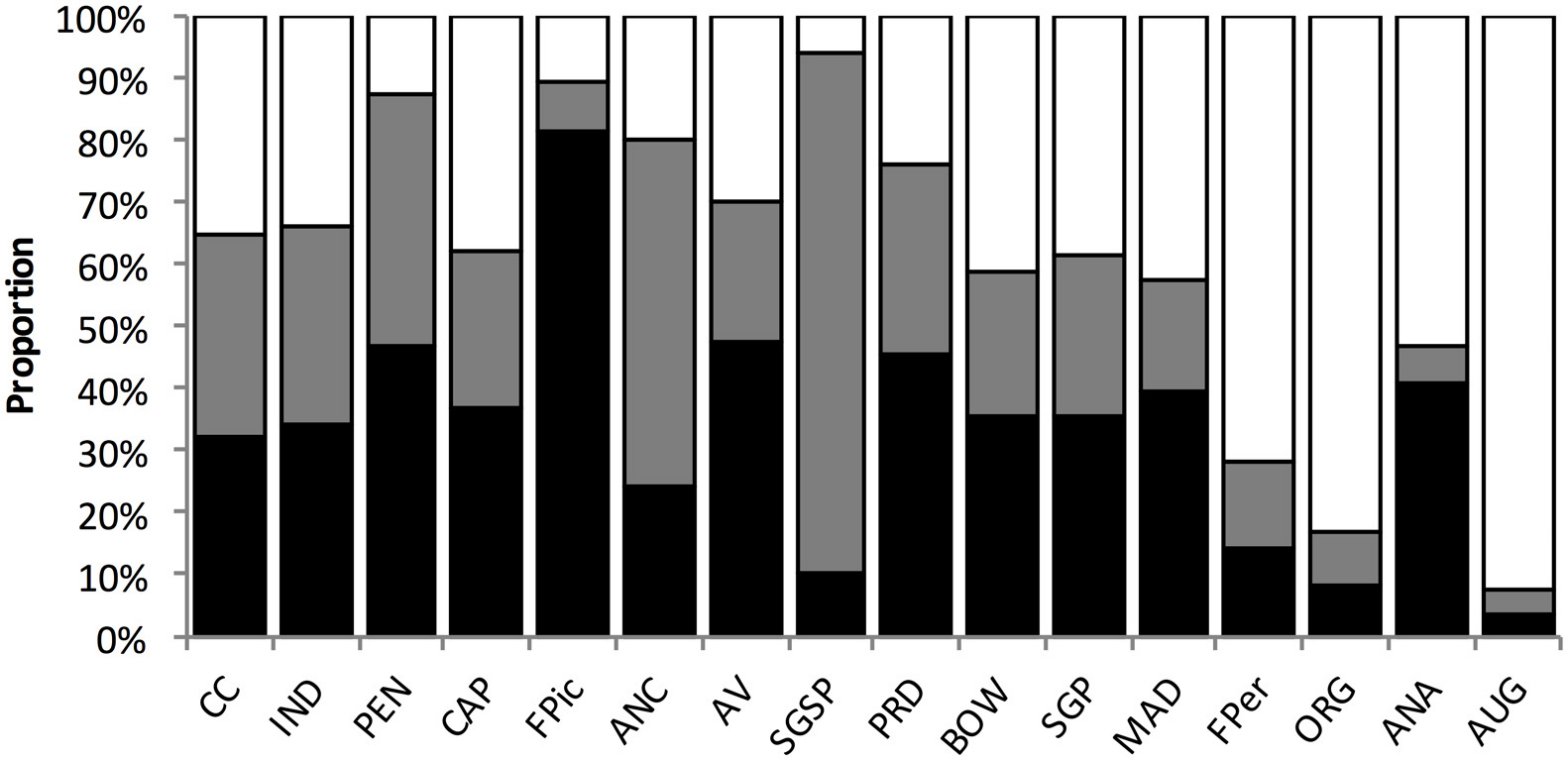
Variation in proportion of three principal components of ghost crab diets calculated from Stable Isotope Analysis in R (SIAR) across the range of beach width (see S1 Table). Prey component coding as follows: Coquina clam (*Donax variablis*) white;, Amphipod (*Talorchestia* spp.) grey;, Mole crab (*Emerita talpoida)* black. Ghost crab isotopic signatures were assessed from muscle tissue collections (N = 3–7 crabs/site) between May 2010 and July 2012.

**Fig 6:**
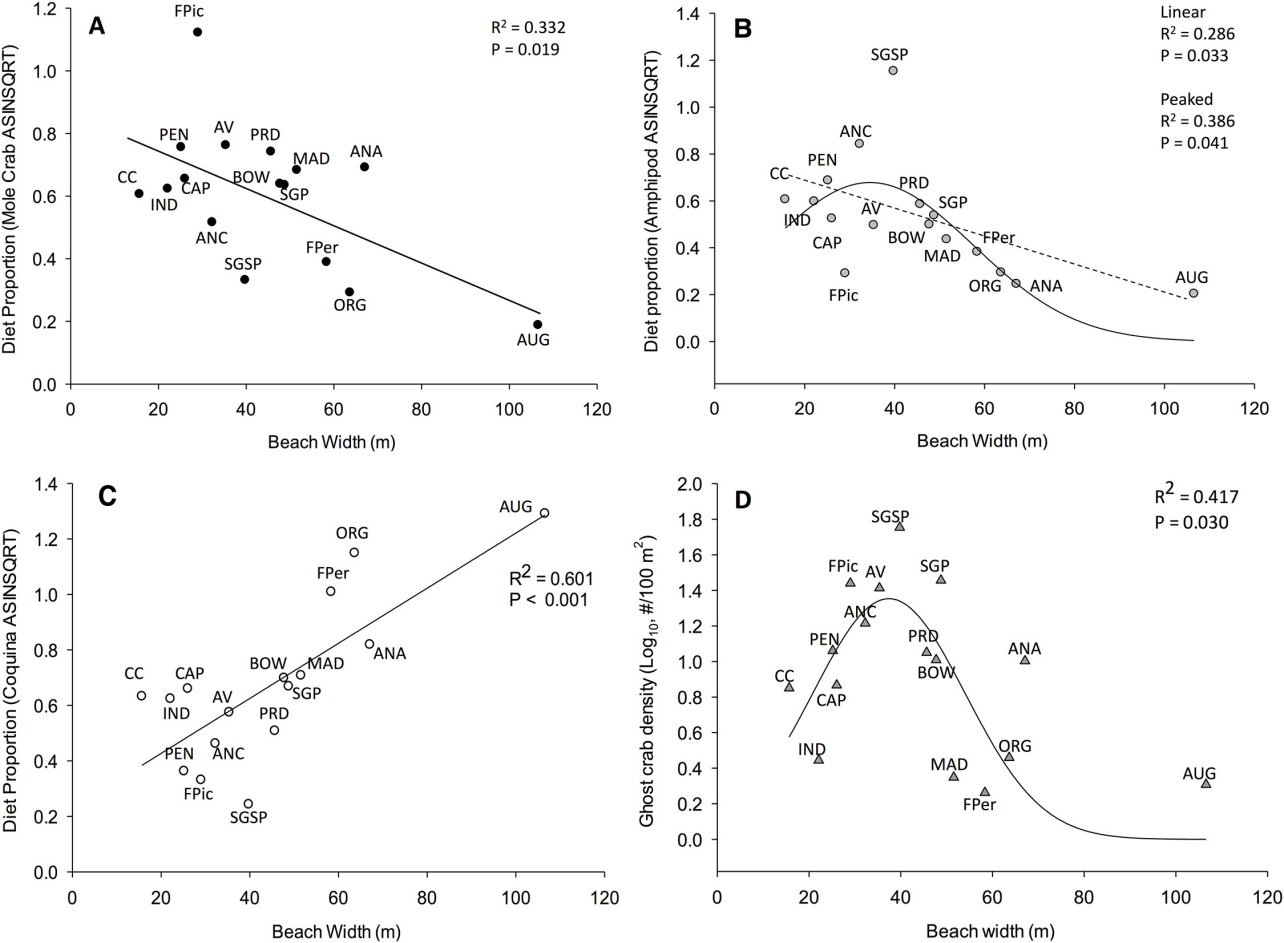
Relationship between beach width (S1 Table) and proportions of dietary components (Fig 5 and S2 Table) for Ghost crabs (*Ocypode quadrata*) determined using Stable Isotope Analysis in R (SIAR). (A) Mole crab (*Emerita talpoida*); (B) Amphipod (*Talorchestia* spp.); and (C) Coquina clam (*Donax variablis*),. (D) Log _10_-transformed mean density of ghost crab (*O*. *quadrata*) in relation to beach width. The relationships between amphipods as a dietary component and Ghost crab density with beach width were modeled using a Gaussian distribution, y = [-0.5 ((x—x_a_)/(b))^2^] (Table 1). Regression coefficient (R^2^) and level of significance (P-value) are indicated for each relationship.

Among all metrics of beach physical setting, only beach width was found to have a significant relationship with contribution of prey types to ghost crab diets. Mean proportion of each of the three principal prey of ghost crabs, determined using stable isotopes and the SIAR mixing model (Fig 5 and S2 Table), displayed significant relationships with beach width using a stepwise regression analysis. However, the nature of the relationship displayed between beach width and proportion of each principal prey in ghost crab diets differed (Fig 6). Specifically, the proportion of mole crabs in ghost crab diets exhibited a significant (F = 6.97, P = 0.019) but negative linear trend with increasing beach width (Table 1—Model 1, Fig 6a). A positive linear relationship between amphipod dietary proportion and physical beach parameters was also well described by beach width (F = 5.61, P = 0.033) (Table 1—Model 2a and Fig 6b). The relationship remained significant (F = 4.10, P = 0.041) when a Gaussian model was applied to the peaked pattern (Table 1—Model 2b and Fig 6b). Proportional contribution of coquina clams to ghost crab diets displayed a positive linear relationship with beach width (F = 21.11, P < 0.001) and this was the strongest relationship revealed in the stepwise regression analyses (Table 1—Model 3 and Fig 6c). Among other beach physical features, only sediment size indicated a marginal (P = 0.091) contribution to the relationship with coquina clams (Table 1—Model 3). Additionally, attempts to describe the relationship between ghost crab density and physical parameters of the beach using a step-wise linear approach failed (Table 1—Model 4a). A Gaussian model best described the relationship between ghost crab density and beach width (F = 4.65, P = 0.030), with ghost crab density peaking at beaches of intermediate width (Table 1—Model 4b and Fig 6d). Again, no other metric of beach physical setting contributed significantly to pattern of ghost crab densities observed across beaches (Table 1—Model 4b).

**Table 1:** Summary of regression analyses for relationships of beach width versus proportions of dietary components (Fig 6a–6c) and mean density of ghost crabs (Fig 6d), beach width versus ghost crab trophic position (Fig 7a) and proportion of dietary items components versus ghost crab trophic position (Fig 7b–7d). A number of non-linear models were utilized when appropriate. Regression coefficient (R^2^), coefficient (Coeff.) and standard coefficient (Std. Coeff.) of independent variables, standard error (Std. Error), F-to-Remove and F-to-Enter: thresholds for inclusion of independent variables in Stepwise regression and level of significance (P-value) are indicated for each relationship.

**Model 1** (Fig 6a)	**Variables in the Stepwise Model**	**R**^**2**^	**Coeff**	**Std. Coeff**	**Std. Error**	**F-to Remove**	**P**
Mole Crab Proportion	Constant		0.860		0.108		
	Width	0.332	-0.006	-0.577	0.002	6.97	***0*.*019***
	**Variables not in the Model**	**F-to Enter**	**P**				
	Slope	0.306	0.589				
	Sediments	0.001	0.981				
	Wrack	0.399	0.538				
**Model 2a** (Fig 6b)	**Variables in the Stepwise Model**	**R**^**2**^	**Coeff**	**Std. Coeff**	**Std. Error**	**F-to Remove**	**P**
Amphipod Proportion	Constant		0.779		0.120		
	Width	0.286	-0.006	-0.535	0.002	5.61	***0*.*033***
	**Variables not in the Model**	**F-to Enter**	**P**				
	Slope	0.310	0.586				
	Sediments	2.722	0.121				
	Wrack	0.248	0.138				
**Model 2b** (Fig 6b)	**Variable/model type**	**R**^**2**^	**df**	**SS**	**MS**	**F**	**P**
Amphipod Proportion	width						
	Non-linear (3 parameter Gaussian)	0.386	2	0.334	0.166	4.10	***0*.*041***
**Model 3** (Fig 6c)	**Variables in the Stepwise Model**	**R**^**2**^	**Coeff**	**Std. Coeff**	**Std. Error**	**F-to Remove**	**P**
Coquina clam Proportion	Constant		0.229		0.107		
	Width	0.601	0.010	0.775	0.002	21.11	***<0*.*001***
	**Variables not in the Model**	**F-to Enter**	**P**				
	Slope	0.031	0.862				
	Sediments	3.299	0.091				
	Wrack	1.510	0.239				
**Model 4a** (Fig 6d)	**Variables in the Stepwise Model**	**R**^**2**^	**Coeff**	**Std. Coeff**	**Std. Error**	**F-to Remove**	**P**
Ghost crab density	None	n/a	n/a	n/a	n/a	n/a	n/a
	**Variables not in the Model**	**F-to Enter**	**P**				
	Width	2.524	0.134				
	Slope	0.031	0.862				
	Sediments	3.299	0.091				
	Wrack	1.510	0.239				
**Model 4b** (Fig 6d)	**Variable/model type**	**R**^**2**^	**df**	**SS**	**MS**	**F**	**P**
Ghost crab density	Non-linear (3 parameter Gaussian)						
	Width	0.417	2	1.339	0.670	4.65	***0*.*030***
	Slope	0.007	2	0.023	0.012	0.05	0.954
	Sediments	0.004	2	0.012	0.006	0.25	0.975
	Wrack	0.219	2	0.703	0.351	1.82	0.201
**Model 5** (Fig 7a)	**Variables in the Stepwise Model**	**R**^**2**^	**Coeff**	**Std. Coeff**	**Std. Error**	**F-to Remove**	**P**
Ghost crab	Constant		3.887		0.228		
Trophic Position	Width	0.592	-0.021	-0.770	0.005	20.35	***<0*.*001***
	**Variables not in the Model**	**F-to Enter**	**P**				
	Slope	0.468	0.505				
	Sediments	0.173	0.684				
	Wrack	1.329	0.268				
**Model 6** (Fig 7b)	**Variable/model type**	**R**^**2**^	**df**	**SS**	**MS**	**F**	**P**
Ghost crab	Mole crab Proportion						
Trophic Position	Linear model	0.245	1	1.332	1.332	4.54	0.051
	Non-linear (2 parameter Hyperbola)	0.356	1	1.938	1.938	7.75	***0*.*015***
**Model 7** (Fig 7c)	**Variable/model type**	**R**^**2**^	**df**	**SS**	**MS**	**F**	**P**
Ghost crab	Amphipod Proportion						
Trophic Position	Linear model	0.623	1	3.175	3.175	21.44	***<0*.*001***
**Model 8** (Fig 7d)	**Variable/model type**	**R**^**2**^	**df**	**SS**	**MS**	**F**	**P**
Ghost crab	Coquina clam Proportion						
Trophic Position	Linear model	0.315	1	1.711	1.711	6.43	***0*.*024***

**Fig 7:**
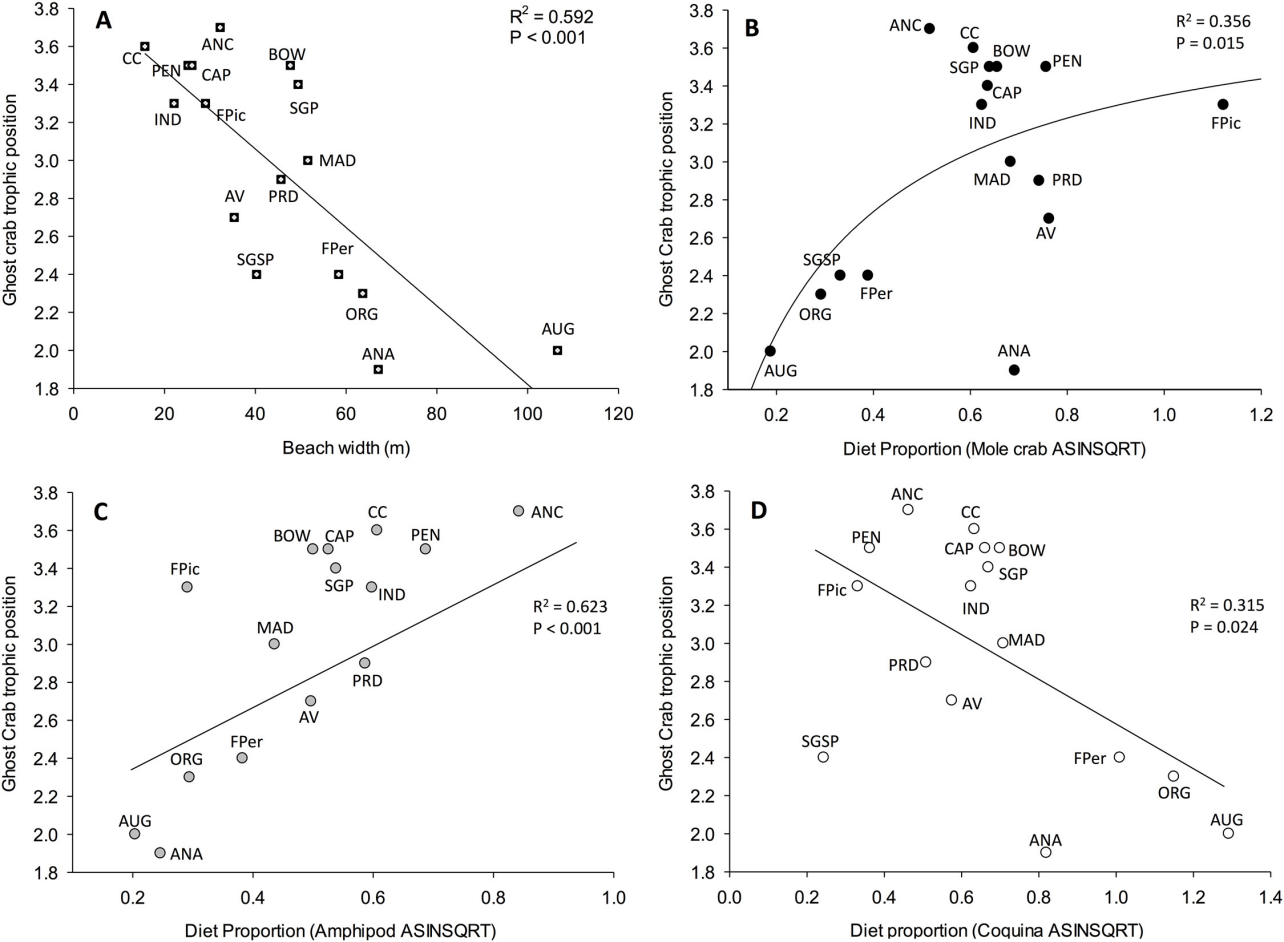
Relationship between: (A) ghost crab trophic position and beach width and B-D) ghost crab trophic position and dietary proportion of three principal prey items (S2 Table). Principal prey are: (B) Mole crab, *Emerita talpoida*; (C) Amphipod, *Talorchestia* spp. (data point for SGSP excluded from regression, see results and discussion); and (D) Coquina clam, *Donax variablis*. The dietary component of *E*. *talpoida* was modeled using an exponential saturating hyperbola, y = a (1-e^-bx^). Regression coefficient (R^2^) and level of significance (P-value) are indicated for each relationship.

Trophic position (TP) of ghost crabs ranged from 3.7 (ANC, beach width = 32 m) to 1.9 (ANA, beach width = 67 m). The stepwise regression procedure revealed that beach width was the single physical beach parameter that contributed to a useful model description with TP decreasing significantly (F = 20.35, P < 0.001) as beach width increased (Table 1—Model 5 and Fig 7a). Slope, sediment size and wrack biomass did not add predictive power (P > 0.268) (Table 1—Model 5). Significant relationships were found when examining ghost crab TP and the proportion of each principal prey. Highest ghost crab TP was generally associated with higher use of mole crabs (hyperbola model, F = 7.75, P = 0.015) (Table 1—model 6 and Fig 7b) and/or amphipods (F = 21.44, P < 0.001) (Table 1—model 7 and Fig 7c). However a significant negative relationship between ghost crab TP and the proportion of coquina clams in their diets was recorded (F = 6.43, P = 0.024) (Table 1—model 8 and Fig 7d).

## Discussion

The examination of food web structure and dynamics provides extremely fertile ground for exploring critical links between biodiversity, functionality, and persistence of ecosystems in the wake of environmental variation and disturbance [1, 3, 75, 76]. The “dynamic food web approach” sets out to document variation in interactions of a specific food web (e.g., lake, rocky intertidal, sandy beach) examined across an environmental/physical gradient. This approach builds on the expectation that spatial and temporal heterogeneity in selected ecosystems fundamentally alters resource availability, trophic dynamics, and community structure. Additionally, this approach suggests that these empirical patterns of change can be used to predict the implications of human impact on ecosystems [7, 9, 12, 33].

Here we explored whether diet and trophic position of a principal predator of sandy beaches—the Atlantic ghost crab—living in an aquatic-terrestrial coupled system display a strong relationship to changes in habitat morphology created by a combination of physical parameters driven principally by beach width (Fig 1). We *a priori* established a working model (Fig 2) that predicted the availability of prey resources and ghost crab dietary composition along a gradient of beach width and associated micro-habitats important to prey. Over the 16 beaches examined, we found evidence to support the predicted relationships from the working model across the range of beach morphology. Although most beaches contained populations of all three principal ghost crab prey, the variation in habitat conditions displayed with increasing beach width were accompanied by quantifiable shifts in ghost crab diet and trophic position. Furthermore, the observed patterns of trophic position of ghost crabs suggest that these predators may augment diets of principal prey in fundamentally different ways along the continuum of beach sizes. Below we discuss these findings in more detail.

Shifts in the diet of consumers as a result of variation in abundance of food resources have been reported in trophic studies from diverse settings including lakes, islands and seagrass beds [9, 77, 78]. In accordance with our working model (Fig 2), examples of dietary shifts were observed for Atlantic ghost crabs as physical features of habitats varied along a gradient of increasing beach width. For most beaches, macroinfauna from swash zones composed a large proportion of ghost crab diets. As beach width increased, macrofaunal resource availability in the swash shifted from mole crabs to coquina clams. We observed the highest occurrence of mole crabs in ghost crab diets at narrow (< 30 m) beach sites best represented by FPic and PEN. These narrow sites also have relatively steep swash zones and coarse sediments which are preferred by mole crabs [79, 80]. Ghost crabs from some narrow sites which supported high mole crab densities (CC, IND) did not display a high proportion of mole crabs in their diets perhaps indicative of reduced resource accessibility. Specifically, ghost crabs are poor swimmers and may avoid some feeding areas if strong wave action is present. In contrast, the highest occurrence of coquina clam resources in ghost crab diets was recorded at wide (> 50 m) beach sites best represented by FPer, ORG and AUG, which hosted moderately-sized sediments and shallow slopes known to be preferred by coquina clams [79, 80]. Interestingly, some intermediate width beaches also had among the smallest sediment grain sizes (e.g. median grain size ANC = 0.18, MAD = 0.25) in the swash zone observed across all sites. These finer sediments may reduce the suitability of these beaches for both mole crabs and coquina clams by hindering their feeding and burrowing [28, 81, 82] and may underlie an increased contribution of alternate or supplemental prey (i.e. amphipods) to the ghost crab diet.

New information on ghost crab diets emerged from the examination of beach morphology and levels of macrophyte wrack accumulation. We recorded a peak in amphipod resources in ghost crab diets at beaches of intermediate (30–50 m) width best represented by ANC and SGSP. This appears to be strongly linked to the high macrophyte wrack biomass supporting semi-terrestrial amphipods [33, 35]. Again, this diet shift may reflect a lower density of swash zone prey as fine sediments in the swash zone may be less suitable habitat for mole crabs and coquina clams [28, 81, 82]. However, the quality of the wrack material is of interest as amphipods are more abundant on newly deposited (i.e. moist) banks of macrophytes where intense metabolism exists [32] when compared to drier, older sources in low abundance [35, 64, 83, 84]. Thus, the low levels of macrophyte wrack biomass at narrow and wide beaches should provide less available habitat for semi-terrestrial amphipods [33, 34] than wrack on intermediate beaches. It should be noted that some authors have found weak correlations between amphipod populations and wrack [16]. In addition, low wrack biomass is likely of minor importance to mole crabs and coquina clams which shelter within moderate to coarse sized sediments at the ocean-beach interface with their particulate organic food mainly delivered by waves and tides [57, 80–82]. The tendency of intermediate beaches to “retain” high amounts of wrack and associated amphipods is likely due to a combination of moderate slope and tidal extent which leads to elevated amounts of wrack in a “favorable” position on the beach. Wrack stranded on narrow beaches may rapidly be swept off by frequent, high energy waves, and wrack stranded high on the beach at wide sites may become desiccated due to the extensive tidal recession period; these two opposing conditions have the potential to reduce the food quality or provision of moist shelter for amphipods. Interestingly, the highest ghost crab densities were found at SGSP where the diet was largely based on amphipods (> 80%) with extremely low abundances of swash zone clams and mole crabs recovered from cores at this site. SGSP is a large State park with over 300 documented species of birds including a number of migratory species (plovers, sandpipers, and turnstones) which may readily deplete the macro-invertebrate resources of the swash zone periodically and directly compete with ghost crabs at times. Again, intense metabolism should support organismal growth where moist wrack accumulates [32]. As such, nocturnally active and abundant amphipods in the wrack will be a critical resource to resident ghost crabs. Macrophyte wrack has been reported to serve as a subsidy for terrestrially-based food webs [12, 29, 30] but here we show that the wrack, principally at intermediate width beaches, also provides food resources for ghost crabs—an important and widespread consumer with a marine origin.

A unique set of results emerged from our investigation of ghost crab trophic position (TP) across beaches with varying morphology. Changes in access to micro-habitats and associated resources have been documented to alter consumer trophic position and degree of omnivory in other systems [9, 27] and this too appears to be displayed within sandy beaches. Specifically, in contrast to our earlier prediction of a consistent TP for ghost crabs across the range of habitat morphology, we found an inverse relationship between ghost crab trophic position and increasing beach width. On narrow beaches the TP values for ghost crabs were elevated relative to the expected TP value of a secondary consumer (3.0), while on wide beaches TP values were generally less than 3.0. Furthermore, the patterns of trophic position align with changing proportions of principal macrofaunal prey in ghost crab diets. Notably, trophic position was elevated with increasing use of amphipods. A single exception was a lower TP value of 2.4 at SGSP despite a very high contribution of amphipods to ghost crab diets having been recorded. More strikingly, a shifting dominance of mole crabs to coquinas in ghost crab diets mirrored increases in beach width as well as a decrease in trophic position. The reduced TP values may be explained by a proportionally increasing amount of basal resources such as microalgae or detritus acquired by ghost crabs as they consume clams, including any with stomach contents. In contrast, ghost crabs may avoid ingesting stomach contents of the comparatively larger mole crabs as they can select muscle tissue [37] which may be difficult when consuming either the smaller amphipods or coquinas. In addition, ghost crabs may supplement diets by microphagous depositing feeding when beaches become wider and less physically dynamic [49, 85]. Higher values of TP (> 3) of ghost crabs from some beaches may indicate the use of carrion of secondary and tertiary consumers or eggs (e.g. fish, marine turtles, birds) [11, 50, 86]. Overall, our findings reveal that ghost crabs appear to supplement macro-invertebrate-based diets with carrion or basal resources on a site specific basis and therefore display true omnivory and an inherent flexibility of a generalist consumer living in a highly dynamic ecosystem [38].

Despite the multiple lines of evidence we used to erect a working model (Fig 2) that matches dietary patterns of ghost crabs to beach features and relative abundances of food resources, there were a number of circumstances when the model was not fully applicable. In fact, while some beaches displayed close agreement with the working model, we also found examples of overlapping dietary composition for ghost crabs collected from a subset of narrow, intermediate and wide category beaches. The reasons for these contrasting relationships between diets and beach morphology may be related to a number of factors which merit additional scrutiny. The high variability in natural physical conditions at sandy beaches is well known and even a single large cyclonic storm can completely alter the morphology of a beach in a short time [5, 52]. Thus the ‘average’ physical configuration of a single beach may not necessarily be encountered over limited sampling intervals and the number of beaches included. High variability in the production of wrack and macro-invertebrates within a beach has been amply noted by others [5, 87–89]. However, our sampling across the peak activity period of all included consumers (April–October) and over multiple years (2010–2012) should provide a reasonably good description of relative changes in consumers, resources and the physical environment across our gradient [5, 57–59]. Better documentation of ghost crab behavior and accessibility to resources, including the consumer’s ability to forage in strong wave surge, fine or densely-packed sediments and macrophyte wrack is desirable; such information will improve our working model and help evaluate the assumption that ghost crabs have access to all food resources on every beach. Additionally, investigations of ghost crab feeding in response to the presence of their own native (herons, gulls, raccoons) and introduced predators (foxes, feral cats, dogs) as well as competitors (plovers, sandpipers, and turnstones) may be informative [11, 31, 43, 85, 86, 90] and may help explain the strong patterns of highest ghost crab density being recorded on beaches of intermediate width reported here. However, despite these limitations, our study provides new details on diets and trophic position given our use of longer-term, integrated measures of ghost crab diets (stable isotopes) and a dynamic food web approach. This may better allow the relative “sampling efforts” of the ghost crabs themselves to reveal the most regular pattern of feeding across the diversity of habitat morphologies where they live [43, 47, 62, 63].

Our results offer support for a functional response in the trophic architecture of sandy beaches across well-known and changing beach morphologies [19–21] through the lens of a globally common food web compartment (ghost crabs—principal macro-invertebrate prey). We also reveal the details of ghost crab diets at specific beaches which they inhabit through the integration of prey tissues (i.e. stable isotopes). The results from our "dynamic food web approach" also offer a basis for evaluating how coastal ecosystems should respond to a variety of anthropogenic impacts [68, 91]. Human—related activities on sandy beaches include: (1) beach grooming which removes stranded macrophytes and carrion; (2) beach re-nourishment which leads to changes in beach width and sediment characteristics, (3) introduction of non-native or feral mammals as novel predators and (4) pedestrian and vehicular traffic which damages beach/dune vegetation, compacts sediments and disturbs wildlife. All may contribute to measureable alterations in micro-habitat conditions, prey availability and ghost crab abundance [52, 90, 92–95]. Human impacts on beach trophic structure might likely be comparatively low on narrow beaches where mole crabs in the swash dominate ghost crab diets and accumulation of wrack is minimal. However, beach grooming or removal of macrophyte wrack may lead to reduced availability of amphipod prey on beaches of intermediate width, where our findings indicate that wrack abundance and amphipod contribution to diets of ghost crabs was greatest. In addition, while diets of ghost crabs from our study sites were typically dominated by macro-infaunal prey, seasonal consumption of carrion washed onto beaches may also contribute to ghost crab diets [11]; we noted stranded urchins as an important source of carrion from preliminary studies at our Cayo Costa (CC) site (Tewfik, unpublished). Therefore the removal of carrion via beach grooming may seriously impact the omnivorous diet and populations of ghost crabs at some locations. Beach re-nourishment via deposition of sediments from offshore locations, a response to beach erosion, may contribute sediments with finer/ coarser grain size than that of the recipient sandy beach and thereby altering micro-habitats and reducing swash zone macro-infaunal populations as well as organisms at higher trophic levels [96, 97]. The introduction of non-native/feral predators may impact ghost crabs directly or through changes in access to ephemeral food resources (e.g. carrion) on some beaches [11, 90]. Finally, the disturbance of ghost crab populations as a result of pedestrians and vehicles, especially on intermediate and wide beaches [52, 98, 99], may provoke changes in food web structure and trophic dynamics given that ghost crabs serve as prey to higher level consumers (herons, gulls, raccoons) which, in turn, couple between other large food webs—coastal forests, dunes and sub-tidal systems [11, 46, 47, 86, 100]. Thus, similarly to a report by Tunney et al. (2014) on shifting trophic interactions by lake dwelling fish in response to changes in prey accessibility, we suggest that anthropogenic impacts on sandy beaches might be identified by comparing consumer diets across an environmental gradient of habitat morphology.

## Supporting Information

S1 Table Physical features of beaches included in the study ordered by increasing beach width. See methods for details of sampling protocol. Sediment = median grain size; % wrack composition = % of dry weight (g) biomass: SG = Seagrass (Th = *Thalassia testudinum*, Syr = *Syringodium filiforme*, Hal = *Halodule wrightii*); MA = Macroalgae and TerrP = Terrestrial plant. Detailed constituents of seagrass wrack are listed when seagrass was greater than five percent of total wrack.
(XLSX)

S2 Table Mean isotopic signatures (δ^13^C, δ^15^N) used to determine diet mixture results from SIAR for beaches included in this study and ordered by increasing beach width at sites (Codes and location see Fig 3). Basal resource isotopic signatures are for dominant forms (SG = Seagrass, Th = *Thalassia testudinum*, Syr = *Syringodium filiforme*, MA = Macroalgae, Sarg = *Sargassum* sp., two species indicates a mixture of SG materials within beach wrack (see S1 Table). *Halodule wrightii* (SG) was not considered given its minor contribution to wrack (see S1 Table). % of GC diet = Percent contribution to ghost crab (GC) diet. Ghost crab (*Ocypode quadrata*) major prey items (principal resources): Coquina clam (*Donax variabilis*), Mole Crab (*Emerita talpoida*), Amphipod (*Talorchestia* spp.). Ghost crab trophic position (GC TP) determined using the following equation: TP_GC_ = [(δ^15^N_GC_− δ^15^N_RES_)/2.3] + 2, where δ^15^N_GC_ represents the mean signature (N = 3–9 indiv.) of ghost crabs; δ^15^N_RES_ represents the weighted mean signature of the three principal resources; and 2.3 is one trophic level change in aquatic environments (See methods for details).
(XLSX)
